# Green Olive Browning Differ Between Cultivars

**DOI:** 10.3389/fpls.2019.01260

**Published:** 2019-10-08

**Authors:** Shiri Goldental-Cohen, Iris Biton, Yair Many, Sivan Ben-Sason, Hanita Zemach, Benjamin Avidan, Giora Ben-Ari

**Affiliations:** Institute of Plant Science Volcani Center, ARO, Bet-Dagan, Israel

**Keywords:** *Olea europaea*, browning, table olive, mechanical harvest, germplasm collection

## Abstract

Currently, table olives, unlike oil olives, are harvested manually. Shortage of manpower and increasing labor costs are the main incentives to mechanizing the harvesting of table olives. One of the major limiting factors in adopting mechanical harvest of table olives is the injury to fruit during mechanical harvest, which lowers the quality of the final product. In this study, we used the Israeli germplasm collection of olive cultivars at the Volcani Institute to screen the sensitivity of many olive cultivars to browning in response to injury. The browning process after induced mechanical injury was characterized in 106 olive cultivars. The proportional area of brown coloring after injury, compared to the total fruit surface area, ranged from 0 to 83.61%. Fourteen cultivars were found to be resistant to browning and did not show any brown spot 3 h after application of pressure. Among them, there are some cultivars that can serve as table olives. The different response to mechanical damage shown by the cultivars could be mainly due to genetic differences. Mesocarp cells in the fruits of the sensitive cultivars were damaged and missing the cell wall as a result of the applied pressure. The cuticles of resistant cultivars were thicker compared to those of susceptible cultivars. Finally, we showed that the browning process is enzymatic. We suggest cuticle thickness as an indicator of table olive cultivars suitable for mechanical harvest. A shift to browning-resistant cultivars in place of the popular cultivars currently in use will enable the mechanical harvest of table olive without affecting fruit quality.

## Introduction

Worldwide production of table olives has risen steadily over the last 30 years from 0.95 million tons in 1990 to 2.9 million tons in 2018. Consumption of table olives has not been far behind, rising from about 1 million tons worldwide in 1996 to 2.7 million tons in 2018 (IOOC, 2019).

Oil olives are harvested when a light green to purple coloration is achieved and fruit softening begins. In contrast to this, most table olives are harvested toward the end of the mesocarp development stage (before physiological maturation), when fruit detachment force (DF) is very high. This hinders fruit harvesting and increases the effort necessary in carrying out this task. Currently, table olives are still harvested manually. Shortage of manpower for hand harvesting and increasing labor costs are the major reasons for mechanizing the harvesting of table olives. At present, manual harvesting is the most expensive phase of table olive production (Ferguson et al., 2010; Zipori et al., 2014). The two major obstacles involved in shifting from manual to mechanical harvesting of table olives are harvesting efficiency and fruit injury.

Abscission agents currently in use for lowering fruit DF before harvest, as a pretreatment for mechanical harvesting of oil olives, are ethylene-releasing compounds such as ethephon (2-chloroethyl phosphonic acid), which achieve consistent fruit loosening. However, ethephon application also resulted in attendant leaf loss coincident with fruit loosening (Hartmann et al., 1970; Martin, 1994; Burns et al., 2008). Recently, we have shown that treating olive-bearing trees with a combination of ethephon and antioxidants reduces DF fruit without weakening that of the leaves (Goldental-Cohen et al., 2017). However, mechanical harvesting technologies used for harvesting oil olives cannot be applied to table olives, due to the high percentage of fruit damaged during mechanical harvesting (Gambella et al., 2013; Jiménez-Jiménez et al., 2013b; Zipori et al., 2014; Jiménez et al., 2016).

As in other fruits, bruising of olives causes changes in the fruit skin color (Samim and Banks, 1993; Conway et al., 2003). The current food industry demands high-quality products. In small fruits such as olives, classification is based on visual characteristics such as the color of the skin or the presence of external defects. Currently, most factories use automatic machines for sorting defective olives by color, and defective fruits are rejected and cause considerable financial loss (Diaz et al., 2000; Diaz et al., 2004; Garrido Fernández, 2006).

Olive fruit is characterized by a thin exocarp surrounding fleshy mesocarp tissue, which makes up the edible portion of the table olive.

The damage caused by impact during mechanical harvesting of olives appears as brown spots on the fruit’s exterior (Castro-Garcia et al., 2009). The browning process begins with a darkening of a section of the green color on the olive fruit surface. After a while, depending on the severity of the impact, the browning spreads over the exocarp and the mesocarp as well and can even affect the endocarp (Jiménez et al., 2011; Jiménez-Jiménez et al., 2013b). The browning process in olive, as in many other fruits, is produced by oxidation of phenolic compounds by different enzymes, particularly polyphenol oxidase (PPO) (Segovia-Bravo et al., 2007; Segovia-Bravo et al., 2009; Segovia-Bravo et al., 2011). Susceptibility to browning in other fruits was reported to be dependent on multiple factors such as fruit size, fruit shape, firmness, cell wall strength, and others (Studman, 1997; Grotte et al., 2000; Berardinelli et al., 2005; Bollen, 2005). Produce type and cultivar differences account for most of the differences in browning susceptibility. However, it is still not yet clear which factors mostly contribute to the potential of susceptible cultivars to bruising (Opara and Pathare, 2014). In olives, susceptibility to browning was seen to vary among different cultivars. For example, “Manzanillo,” the main table olive in Israel and in other countries, was shown to be relatively sensitive to browning in response to impact (Jiménez-Jiménez et al., 2013a; Jiménez et al., 2016).

Jiménez et al. (2016) described and quantified anatomical changes in the olive mesocarp in response to bruising in olive fruits after an induced impact. They studied the browning characteristics of the cultivars “Manzanillo” and “Hojiblanca” at two time points, 4 and 24 h after induced impact. In response to the impact, fruits exhibited damaged mesocarp tissue, including ruptured cells, loss of cell wall thickness, and discoloration of the damaged areas. These changes were greater in the more sensitive cultivar, “Manzanillo,” compared to the more resistant cultivar, “Hojiblanca.”

As mentioned above, mechanical harvest of green olives, particularly of table olives, can cause brown spots on the fruits. Rejection of these fruits represents financial loss to the farmer. Pre-harvest treatment of olives by an anti-browning agent has been very limited to date, and the inhibitory effect of anti-browning agents on reducing PPO activity was found to be cultivar-dependent (Mohsenabadi et al., 2017). Assuming that cultivar susceptibility is the main factor contributing to browning in response to impact, large scale screening of various cultivars has not been performed, neither the factors causing some cultivars to be more resistant than others to browning have been indicated.

The aim of this study was to evaluate the variation in susceptibility to browning among olive cultivars in order to identify those more resistant and to understand the main factors influencing browning resistance. Olive fruits from 106 olive cultivars in the Israeli germplasm collection were subject to mechanical injury. Elasticity as well as browning level were characterized for all cultivars at 3 and 24 h after impact. Exocarp cells remained unharmed after the impact even in the more susceptible cultivars. However, mesocarp cells were damaged only in the susceptible cultivars. We suggest that the major factor contributing to the variation in susceptibility to browning among cultivars is the thickness of the cuticle.

## Materials and Methods

### Plant Material

The Israeli germplasm collection consists of 119 cultivars, each represented by three 23-year-old trees growing in an irrigated olive orchard. It is located at the Volcani Center (ARO) in Bet Dagan, Israel (31°58’57.7”N 34°49’47.4”E). This collection includes oil producing strains, as well as table olive cultivars from different geographic origins, and represents the most popular varieties grown around the world.

Once a week, beginning September 1^st^ 2016, we visually screened the olive orchard containing the Israeli germplasm collection, in order to identify cultivars in which fruit color changed from green to yellow indicating the physiological stage suitable for the harvest of table olives. Ten fruits from three trees (3–4 fruits from each tree) of each cultivar showing color change were sampled and the fruit immediately taken for analysis of their response to applied mechanical pressure. Out of the 119 cultivars, 13 bore no fruit; therefore, fruits from only 106 cultivars were analyzed. Damage induction and elasticity evaluation of the 20 cultivars whose response to mechanical pressure registered at the two extremes of the scale (10-resistant and 10-sensitive) were analyzed once again a year later.

### Damage Induction and Elasticity Evaluation

At the time of the table olive harvest, 10 fruits from each cultivar were sampled as described above and immediately subjected to mechanical pressure. Monitoring of pressure and elasticity was done by a force gauge FG-5000A (MRC, Holon, Israel). We used a flat circle pinhead, 8-mm diameter, for both damage induction and elasticity evaluation. Five fruits per cultivar were subjected to damage induction. Each fruit was placed on a metal base and pressed with the force gauge at 3 kg of pressure. Each group of five fruits from the same cultivar was placed in a small plastic tray and incubated at room temperature for 24 h. Fruits were photographed 3 and 24 h after being pressed. Images were collected by Fujifilm FinePix S4300 digital camera (FujiFilm, Ramat Gan, Israel) fixed on a metal stand 10 cm above the fruits. The remaining five fruits from each cultivar were used for evaluating elasticity as follows: fruits were placed on a metal base and pressed 3 mm from the surface of the fruits toward the fruit endocarp. We measured the force needed to move the pinhead 3 mm toward the inner part of the fruit.

### Brown Spot Area

We used Adobe Photoshop software (Onstott, 2012) to calculate the proportion of brown spot resulting from injury to the fruit, relative to the surface area of the fruit. The proportion of brown area was calculated as follow: brown area (cm^2^) = brown pixels/(total pixels/cm^2^). For each cultivar, the average proportion of brown area on five fruits was calculated 3 and 24 h after pressure was applied.

### Fruit Mesocarp and Exocarp Structures

Mesocarp and exocarp structures as well as the cuticle thickness of four resistant and four sensitive cultivars were analyzed. Tissue blocks (approx. 3.0 mm × 1.5 mm × 1.5 mm sizes) were cut revealing the mesocarp and exocarp in longitudinal view. The samples were fixed in 70% ethanol followed by dehydration in gradual ethanol series (90, 95, 100, and 100%). Samples were dried in critical point dryer (K850 Quorom). The dry tissues were attached to a metal stub by double-sided carbon tape and coated with gold palladium (Quorum SC7620 Mini Sputter Coater). Images were taken with a JEOL JCM-6000 benchtop scanning electron microscope (SEM). Analysis was performed using the SEM software. The thickness of the cuticular flange wedged between epidermal cells was measured (Lanza and Di Serio, 2015).

Penetration force was measured using Lutron fruit hardness tester model FR 5105 (Scientific Instrument & Optical Sales, Brisbane, Queensland, Australia) with a 3-mm measuring tip. From each of eight cultivars (four resistant and four sensitive), we tested 20 fruits and measured the force needed to penetrate the exocarp.

### Enzyme Inactivation

In order to test whether the brown spots which appeared in response to the injury caused by mechanical pressure applied to the fruit are enzyme-dependent, we used 10 fruits of the sensitive cultivar “Koroneiki.” All 10 fruits were pressed with the force gauge, and five of these were immediately incubated in a water bath at 65°C for 10 min enclosed in a covered plastic container. All 10 fruits were exposed for 3 h at room temperature then photographed as described above.

### Statistical Analysis

The ratio between brown area and total surface area (after arcsine transformation), elasticity coefficient, thickness of the cuticular flange, and penetration force among cultivars were measured and subjected to one-way analysis of variance (ANOVA) with Tukey–Kramer test using JMP Software (SAS Institute, 2007). The damaged areas as appeared 3 and 24 h after pressing were compared using linear regression and Pearson correlation, in order to derive the regression coefficient (slope) and correlation coefficient. The same analysis was carried out to compare the degree of browning 3 h after pressing and the elasticity coefficient of each cultivar. The degree of browning of the genetic clusters was subjected to a one-way ANOVA and a Tukey–Kramer test. The same procedure was carried out for the elasticity coefficients. The delineation of the various genetic clusters was determined visually according to the dendrogram characterizing the phylogenetic relationships among 119 cultivars of the Israeli germplasm collection, based on a set of 138 SNPs (Biton et al., 2015).

## Results

### Distribution of the Degree of Browning in the Germplasm Collection, as a Result of Mechanical Injury to Olive Fruit

After deliberately injuring fruits of the cultivars composing our germplasm collection, the damaged outer area was measured. Some cultivars proved to be more sensitive to injury than others, and their color began changing from green to brown in the area surrounding the injury (**Supplementary Clip**). The proportional area of the brown spots was measured. Significant variations among cultivars were found. In addition, browning level measured 3 and 24 h after pressing was similar in all cultivars but three. We performed regression analysis between the damaged outer areas 3 and 24 h after pressing. The regression coefficient was 1.033. The proportional area of brown coloring of 3 out of the 106 cultivars, “Dolce de Marocain,” “Memecik,” and “Gemlik,” was much higher 24 h after pressing (54.83, 54.6, and 50.86, respectively) compared to 3 h after pressing (16.94, 18.09, and 19.37%, respectively). However, regression analysis for all cultivars other than the three mentioned above resulted in a regression coefficient of 1.02 and a correlation coefficient of 0.956 (P < 9.34X10^−37^). On this basis, we used the browning level 3 h after pressing to evaluate the variation among cultivars.

**Figure 1:**
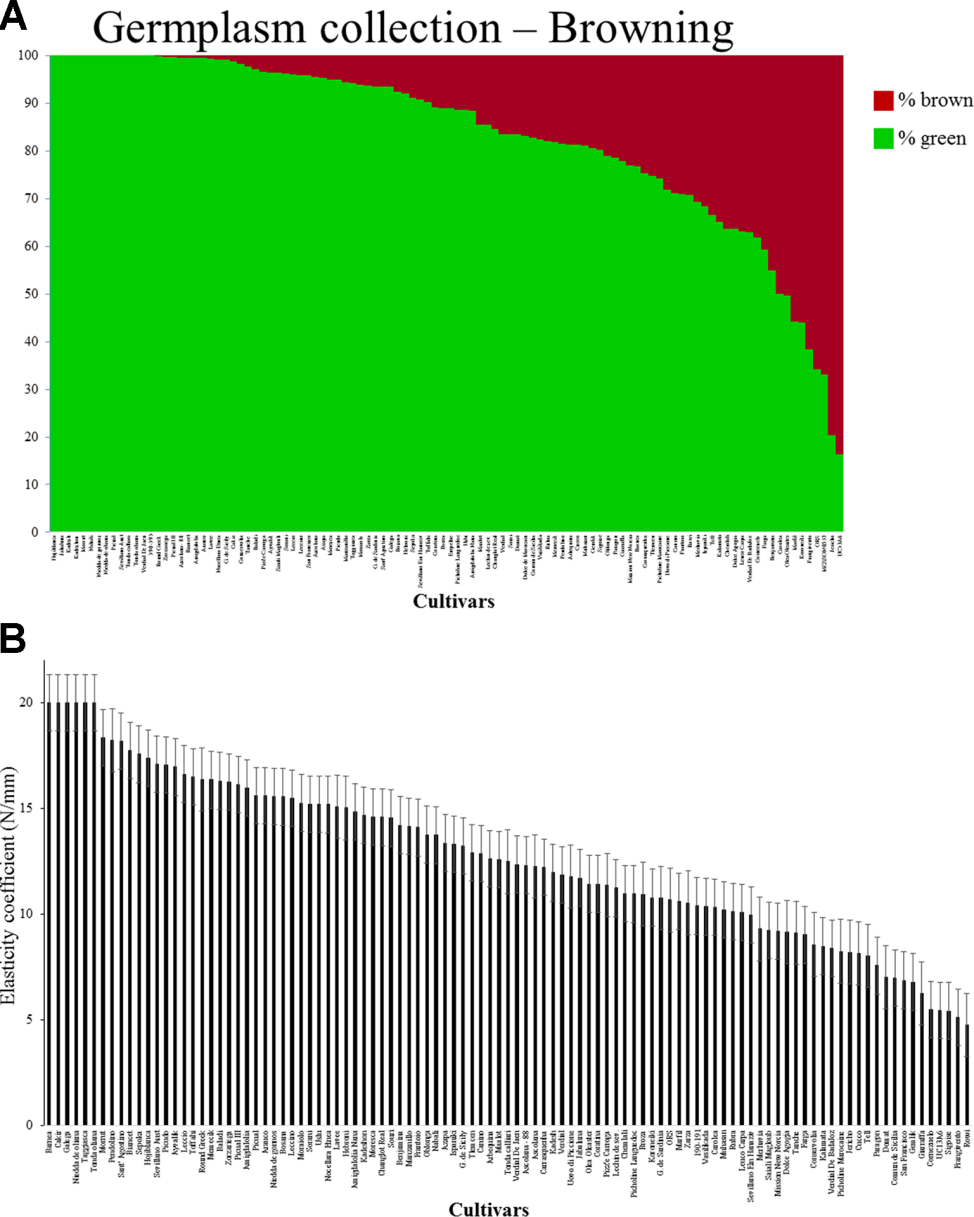
**(A)** Proportional area (expressed in percentage) of the olive surface turned brown as a result of injury. This was measured by a two-dimensional photograph of the fruit of each cultivar. The values were plotted as a column graph. The green and brown column bars at each segment represent the percentage of green and brown colors, respectively, on the surface of the fruit. **(B)** Elasticity coefficient of each cultivar. Error bars represent confidence limits (n = 5; α = 0.05).

The proportional area of brown coloring 3 h after injury, compared to the fruit surface as measured by the two dimensional photograph of the fruit, ranged from 0% in certain cultivars to 83.61% in cultivar UC13A6, with an average of 16.8% (**Figure 1A** and **Table 1**).

**Table 1:** For each cultivar of the germplasm collection, the table describes country of origin, the purpose of the olive cultivar (O, oil cultivar; T, table cultivar; D, dual purpose cultivar), the proportion of brown area 3 h after induced pressure, and the elasticity coefficient (n = 5).

Cultivar	Origin	Purpose	% green	% brown	E(N/mm)
Hojiblanca	Spain	D	100	0	17.38
Jabaluna	Spain	O	100	0	11.71
Kadesh	Israel	T	100	0	11.96
Kadeshon	Israel	T	100	0	14.68
Morrut	Spain	O	100	0	18.33
Nabali	Palestine	D	100	0	13.73
Niedda de gonnos	Italy	D	100	0	15.57
Niedda de oliana			100	0	20.00
Picual	Spain	D	100	0	15.61
Sevillano Aust	Spain	T	100	0	17.09
Tonda calliari	Italy	D	100	0	12.48
Tonda oliana	Italy	D	100	0	20.00
Verdial De Jaen	Spain	O	100	0	12.35
190-191	Israel		100	0	10.39
Round Greek	Greece	T	99.92	0.08	16.36
Zorzariega	Spain	D	99.71	0.29	16.25
Picual III	Spain	O	99.69	0.31	16.12
Ascolana - 88	Italy	T	99.56	0.44	12.31
Biancet	?	O	99.48	0.52	17.73
Amigdalolia	Greece	D	99.47	0.53	15.97
Arauco	Argentina	T	99.43	0.57	15.59
Lavee	Israel	D	99.34	0.66	15.08
Nocellara Etnea	Italy	D	99.24	0.76	15.18
G. de Sicily	Italy	T	99.17	0.83	13.23
Cakir	Turkey	D	98.81	1.19	20.00
Conservolia	Greece	T	98.29	1.71	8.57
Tanche	France	D	97.64	2.36	9.12
Baladi	Lebanon	O	97.12	2.88	16.30
Pizz’e Carroga	Italy	O	96.64	3.36	11.37
Ayvalik	Turkey	O	96.41	3.59	16.96
Saiali Magloub	Tunisia		96.40	3.60	9.24
Sorani	Syria	D	96.26	3.74	15.19
Leccio	Italy	O	95.99	4.01	16.62
Leccino	Italy	O	95.91	4.09	15.48
San Francisco	Italy	O	95.81	4.19	6.87
Ascolana	Italy	T	95.50	4.50	12.26
Azapa	Chile	T	95.41	4.59	13.36
Moresca	Italy	D	94.93	5.07	14.60
Picudo	Spain	O	94.89	5.11	17.06
Manzanillo	Spain	T	94.50	5.50	14.14
Taggiasca	Italy	O	94.22	5.78	20.00
Moraiolo	Italy	O	93.84	6.16	15.26
Zarza	Spain		93.76	6.24	10.54
G. de Sardinia	Italy	T	93.47	6.53	10.78
Sant’ Agostino	Italy	T	93.47	6.53	18.17
Galega	Portugal	O	93.45	6.55	20.00
Bosana	Italy	O	92.43	7.57	15.54
Hebroni	Palestine	T	91.98	8.02	15.02
Sepoka	Israel	T	91.13	8.87	17.56
Sevillano Ein Hanaziv	Israel	T	90.70	9.30	9.97
Toffahi	Egypt	T	90.22	9.78	16.48
Coratina	Italy	O	89.18	10.82	11.43
Broza	Israel	T	89.05	10.95	10.94
Empeltre	Spain	O	88.96	11.04	
Picholine Languedoc	France	D	88.65	11.35	10.95
Uslu	Turkey	D	88.60	11.40	15.19
Amigdalolia Nana	Greece	D	88.34	11.66	14.83
Maalot	Israel	O	85.53	14.47	12.58
Lechin de sev.	Spain	O	85.51	14.49	11.24
Changlot Real	Spain	O	84.62	15.38	14.58
Verdial	Spain	O	83.51	16.49	11.86
Souri	Israel	D	83.47	16.53	14.54
Domat	Turkey	T	83.43	16.57	7.03
Dolce de Marocain	Morocco		83.06	16.94	
Comun de Sicilia	Italy		82.79	17.21	6.98
Vasilikada	Greece		82.48	17.52	10.35
Rubra	?	O	82.14	17.86	10.11
Memecik	Turkey	D	81.91	18.09	16.35
Pendolino	Italy	O	81.52	18.48	18.21
Arbequina	Spain	O	81.36	18.64	12.62
Cucco	Italy	T	81.35	18.65	8.15
Muhasan	Palestine	D	81.08	18.92	10.19
Gemlik	Turkey	D	80.63	19.37	6.79
Sigoise	Algeria	D	80.18	19.82	5.42
Oblonga	USA	O	78.88	21.12	13.76
Paragon	Australia	O	78.67	21.33	7.57
Giarraffa	Italy	T	77.87	22.13	6.25
Mission New Norcia	?	O	76.92	23.08	9.20
Barnea	Israel	O	76.70	23.30	20.00
Carrasquenha	Portugal	T	75.36	24.64	12.23
Tlemcen	Algeria	D	74.78	25.22	12.90
Picholine Marocaine	Morocco	D	74.25	25.75	8.24
Uovo di Piccione	Italy	T	71.83	28.17	11.78
Canino	Italy	O	71.06	28.94	12.87
Frantoio	Italy	O	70.93	29.07	14.10
Rowi	?		70.80	29.20	4.75
Merhavia	Israel	T	69.28	30.72	9.30
Ispaniki	Spain	O	68.40	31.60	13.31
Tell	Algeria	D	66.68	33.32	8.02
Kalamata	Greece	T	65.18	34.82	8.49
Chemlali	Tunisia	O	63.74	36.26	10.97
Dolce Agogia	Italy	O	63.67	36.33	9.15
Leuco Carpa	Italy	O	63.14	36.86	10.09
Verdial De BadaJoz	Spain		62.86	37.14	8.39
Cornezuelo	Spain	T	61.77	38.23	5.48
Farga	Spain	O	59.37	40.63	9.02
Benjamina	Palestine	O	55.01	44.99	14.20
Carolea	Italy	T	50.00	50.00	10.33
Olea Oleaster	Italy		49.65	50.35	11.43
Marfil	Spain	O	44.14	55.86	10.60
Koroneiki	Greece	O	43.94	56.06	10.78
Frangivento	Italy	O	38.30	61.70	5.12
ORS	Australia	O	34.12	65.88	10.67
MCSSON 0517	?		33.19	66.81	
Jericho	Palestine	T	20.28	79.72	8.20
UC13A6	USA	T	16.39	83.61	5.44

In order to evaluate the variation in hardness of different cultivars, we also measured the elasticity coefficient of the fruits of each cultivar. Elasticity coefficient of the various cultivars ranged between 4.75 and 20 N/mm, with an average of 12.67 N/mm (**Figure 1B** and **Table 1**). The degree of browning and the elasticity coefficient of each cultivar was found to be significantly and negatively correlated with a correlation coefficient of −0.526 (P < 1.16X10^−8^). As part of the Israeli breeding program, we tested the average fruit weight and oil content of all cultivars composing the Israeli germplasm collection during 5 consecutive years (data not shown). No significant correlation was found between degree of browning and fruit weight (r = −0.09; P = 0.39) or to oil percentage (r = 0.05; P = 0.65).

**Figure 2:**
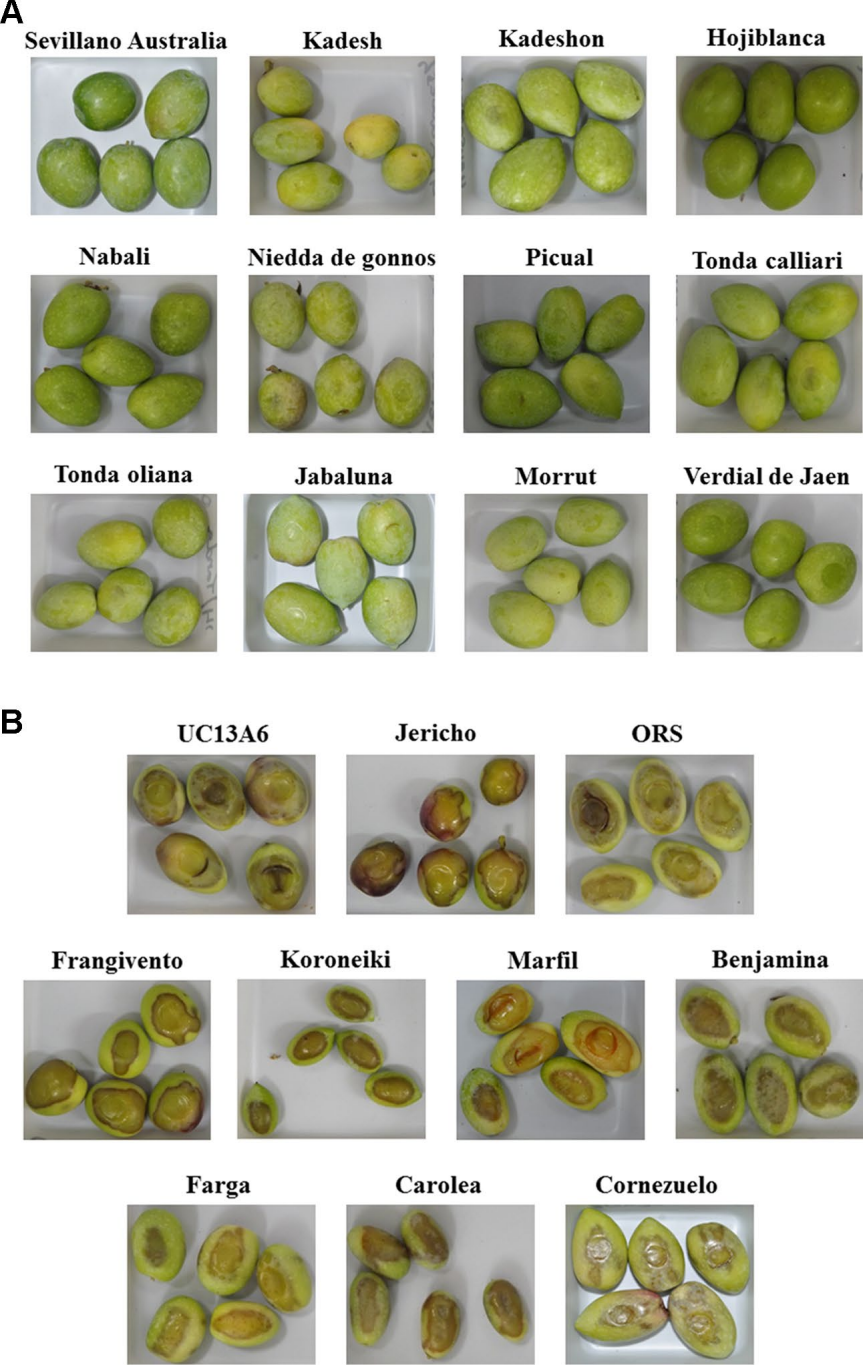
Photographs of five fruits from each of the most resistant **(A)** or susceptible **(B)** cultivars to browning, 3 h after induced mechanical pressure.

Fourteen cultivars were found to be resistant to browning and did not show any brown spot 3 h after application of pressure. Among them, there are several table olives such as the cultivars “Sevillano Australia,” “Kadesh,” and “Kadeshon.” The latter two are table olive cultivars developed as part of the Israeli olive breeding program. Some of the resistant cultivars are dual purpose and can be used either as table or oil olives; these include the cultivars “Hojiblanca,” “Nabali,” “Niedda de gonnos,” “Picual,” “Tonda calliari,” and “Tonda oliana.” Finally, among the resistant cultivars, there are typical oil cultivars such as “Jabaluna,” “Morrut,” and “Verdial de Jaen” (**Figure 2A**). At the other end of our scale, several cultivars exhibited severe browning in response to injury by the 3-kg press. The most sensitive cultivars were the table olive cultivars “UC13A6” and “Jericho,” which showed 83.61 and 79.72% brownings, respectively. In addition, the oil cultivars “ORS,” “Frangivento,” “Koroneiki,” “Marfil,” “Benjamina,” and “Farga” showed browning proportions greater than 40%. Last are the table olive cultivars “Carolea” and “Cornezuelo,” which showed 50 and 38.23% brownings, respectively (**Figure 2B**).

No significant differences were detected between browning degree or cuticle elasticity among cultivars on the basis of their geographical origin.

**Figure 3:**
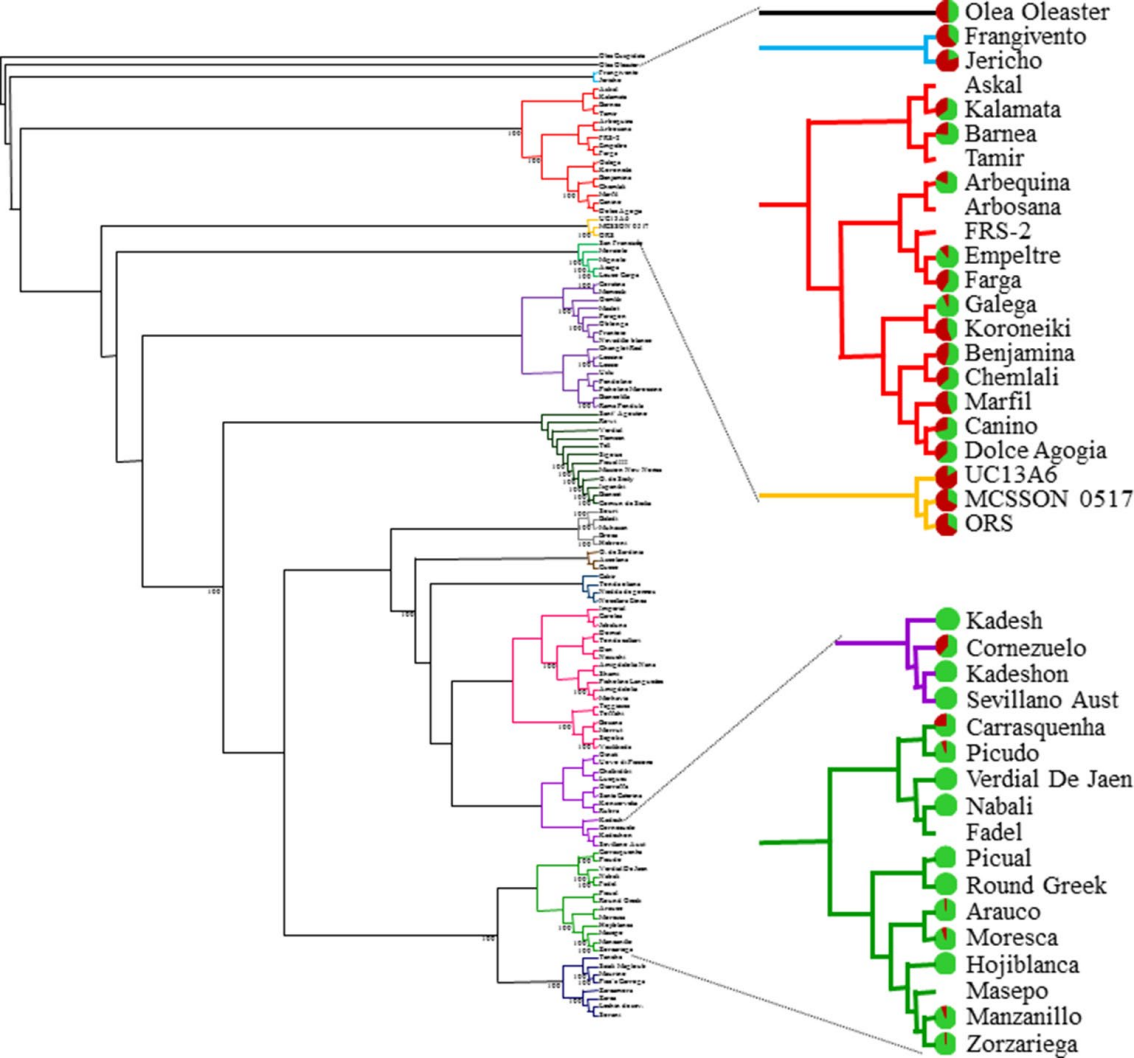
Neighbor-joining tree of 119 cultivars making up most of the Israeli germplasm collection, based on a set of 138 SNPs (Biton et al., 2015). The branches of different clusters appear in different colors. Sub-clusters are enlarged, including the names of the relevant cultivars. For each of the enlarged clusters, the proportion of brown area appears next to the cultivar name, as a pie chart with green and brown areas representing the green and brown proportions of the cultivar fruits as a response to mechanical injury.

The genetic relationships between the 119 cultivars making up the major part of the Israeli germplasm collection had been analyzed by means of a set of 138 SNPs (Biton et al., 2015). According to the dendrogram previously obtained, we clustered the olive cultivars into 13 genetic groups. We then analyzed the differences in browning level and elasticity coefficient among the 13 groups. The average browning level and elasticity coefficient of the cultivars from each of the 13 genetic groups were significantly different (P < 1.17X10^−15^ and P < 0.008, respectively) (**Figure 3**). Many cultivars that are closely related genetically showed similar browning characteristics. For example, the cultivars “UC13A6,” “MCSSON 0517,” and “ORS” are part of the same genetic cluster and show high browning levels (83.61, 66.81, and 65.88%, respectively). The cultivars “Fragivento” and “Jericho” are also genetically close and exhibit high rates of browning (61.7 and 79.72%, respectively). At the other end of the cluster distribution, “Kadesh,” “Kadeshon,” and “Sevillano Australia” are on the same genetic cluster and are all resistant to browning. Although some cultivars showed a unique pattern of browning, unlike other cultivars of the same genetic cluster, such as the browning-sensitive cultivar “Cornezuelo” or the semiresistant cultivar “Galega,” most of the cultivars showed a similar browning pattern as their neighbors, in all clusters (**Figure 3**).

### Fruit Mesocarp Cells Are Damaged by the Press

**Figure 4:**
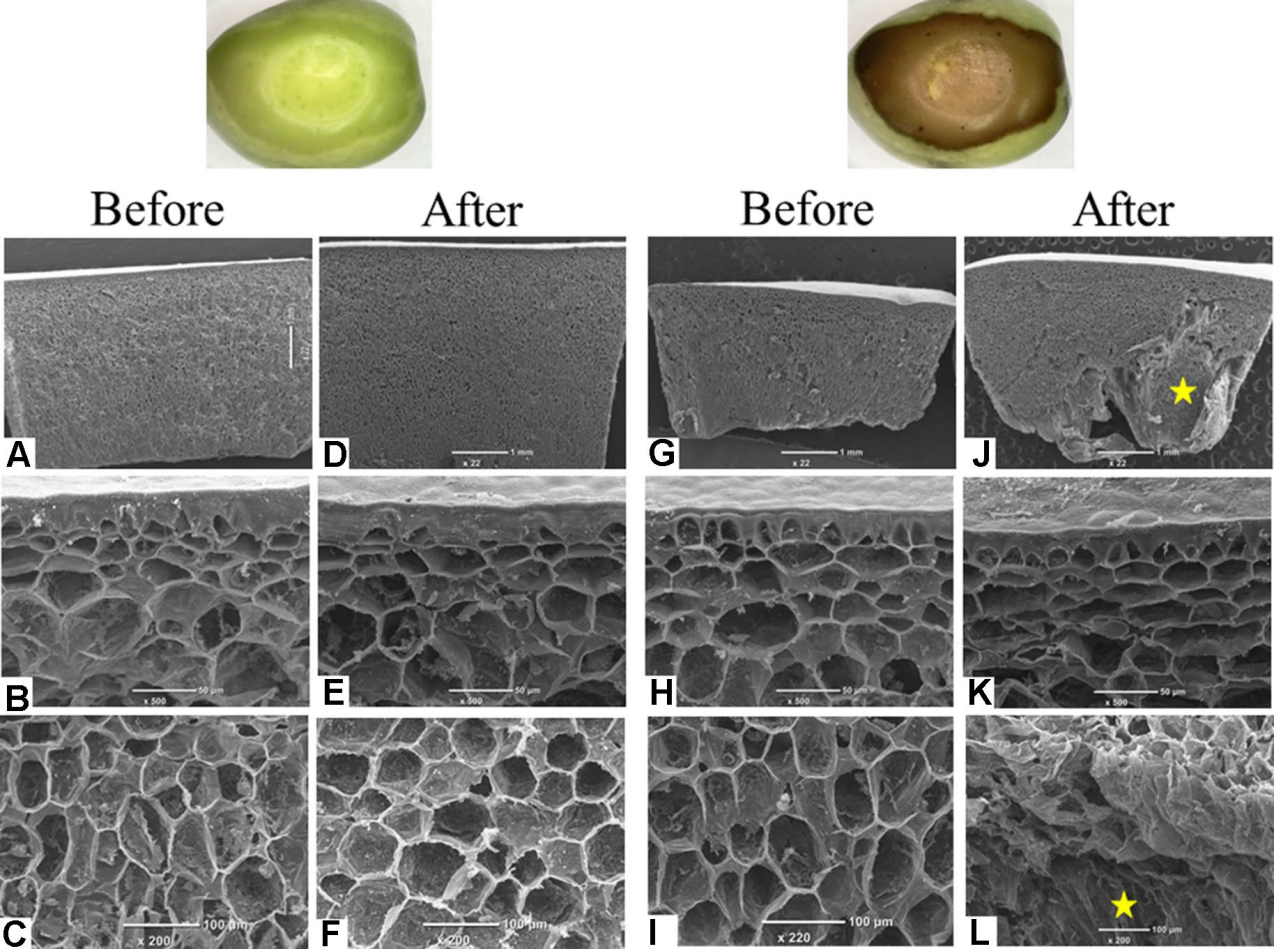
Images before and 3 h after the induced pressure of a representative fruit of browning-resistant (“Nabali”; **A**–**F**) and browning-susceptible (“ORS”; **G**–**L**) cultivars. For each fruit, before **(A**–**C**, **G**–**I)** and after **(D**–**F**, **J**–**L)** exposures to mechanical pressure, three images are presented representing a comprehensive view **(A**, **B**, **G**, **J)**, a zoom in on the exocarp cells **(B**, **E**, **H**, **K**) and a zoom in on the mesocarp cells **(C**, **F**, **L**, **L)**. Mesocarp cell rupture is marked by a yellow star.

In order to understand the relationship between the damage caused by the simulated injury to the fruit and the browning phenomenon, the exocarp and mesocarp cells of the pressed fruits were screened using a scanning electron microscope. Tissue and cell structure were compared after the application of pressure in four sensitive cultivars (‘ORS,” “Carolea,” “Benjamina,” and “Farga”) and four resistant ones (“Hojiblanca,” ‘Morrut,” “Nabali,” and “Kadesh”). Exocarp cells of both groups suffered any damage from the applied pressure. However, some of the mesocarp cells in the fruits of the sensitive cultivars were totally disrupted, and their cell walls were missing as a result of the applied pressure (**Figure 4**).

### The Cuticle of the Resistant Cultivars Is Thicker Than That of Sensitive Cultivars

**Figure 5:**
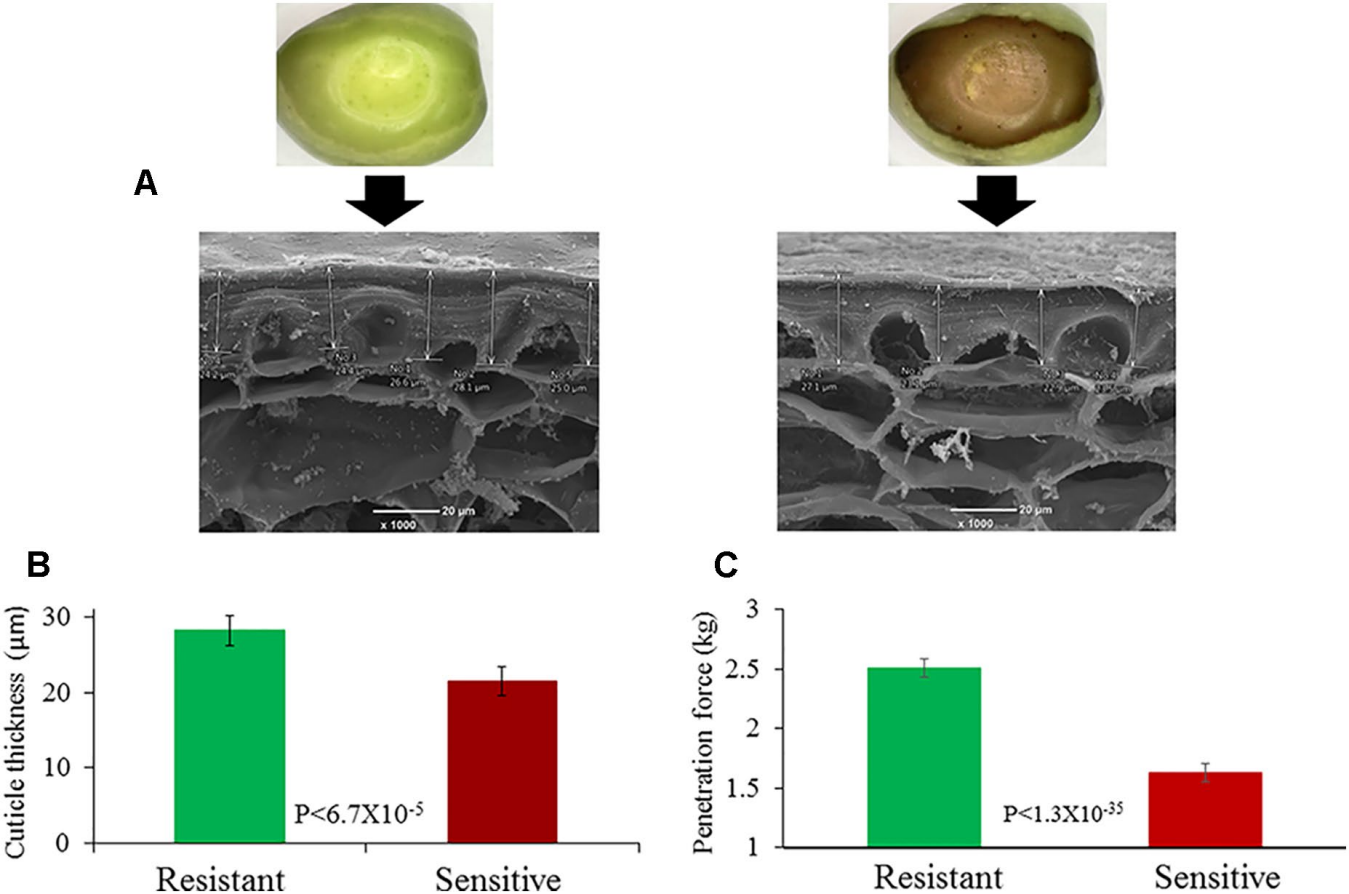
Cuticle thickness measured by SEM. **(A)** Cuticle of a browning-resistant (“Nabali,” left) and a browning-susceptible cultivar (“ORS,” right). **(B)** Average cuticle thickness of browning-resistant and -susceptible cultivars (green and brown columns, respectively). **(C)** Penetration force of browning-resistant and -susceptible cultivars (green and brown columns, respectively). Error bars are confidence limits (n = 5; α = 0.05).

In order to understand the factors which cause some cultivars to be resistant while others are sensitive to pressure, we measured the thickness of the cuticle of fruits from various cultivars. The thickness of the cuticular flange that is wedged between epidermal cells was measured by SEM and compared in fruits sampled from sensitive (“ORS,” “Carolea,” “Benjamina,” and “Farga”) *versus* resistant (“Hojiblanca,” “Morrut,” “Nabali,” and “Kadesh”) cultivars. The average thickness of the cuticle of the resistant cultivars, whose mesocarp cells remained intact, was 28.21 µm, while the cuticle thickness of the sensitive cultivars was 21.49 µm. This difference in cuticle thickness between resistant and sensitive cultivars was significant (P < 6.66X10^−5^) (**Figure 5A**).

We also measured the penetration force of the cuticle. The average penetration force in the resistant cultivars was 2.5 kg, while the penetration force in the sensitive cultivars was significantly less, 1.6 kg (P < 1.3X10^−35^) (**Figure 5B**).

### The Browning Is an Enzyme-Dependent Response

In order to test if the browning response to fruit injury is enzyme-dependent, we sampled fruits from “Koroneiki,” a cultivar of proven sensitivity to pressure. Fruits were pressed by the force gauge, and half the fruits were dipped in boiling water for 10 min, while the other half was incubated at room temperature. The fruits were photographed 3 h after passing through the press. Heated fruits had no brown spots, while control fruits clearly showed large brown spots in response to the applied pressure (**Supplementary Figure 1**).

## Discussion

Mechanical harvesting of oil olives, mostly by trunk shaker or by harvester, is now a common practice in orchards. However, mechanical harvest of table olives is still very limited (Tous and Tous, 2011; Zipori et al., 2014). Recently, we published a study suggesting the use of ethephon and ascorbic acid as a pretreatment to facilitate mechanical harvest of green table olives (Goldental-Cohen et al., 2017). This pretreatment was shown to decrease the DF of the fruits without causing defoliation. However, in order to enable mechanical harvest of table olives, it is also necessary to prevent external fruit color change or browning. In this study, we used our germplasm collection to screen for browning-susceptible and -resistant cultivars. The various olive cultivars differ in their degree of browning in response to injury. Fruits of some cultivars are completely resistant and remained green 24 h after impact, while others developed a large brown spot around the point of impact. We also found that the browning process in response to impact begins with damage to the mesocarp cells while a thick cuticle inhibits this process.

### Some Cultivars Are Resistant to Browning

In this study, the parameter used to evaluate the differences among cultivars was the two-dimensional proportion of the brown area around the impact point in relation to the surface area of the whole fruit. Although this parameter may be inaccurate in evaluating total damage to the fruit, because it takes into account only two dimensions, it should provide enough information to be used as a fast and easy method for screening commercial quantities of fruit. Using this parameter, we were easily able to screen the damaged area of each of 5 fruits from 106 cultivars for a total of 530 fruits. Jiménez et al. (2016) quantitatively defined bruising damage to olives by 11 parameters. Of these, they recommended the use of three parameters as the most discriminant, the easiest to assess and the most useful for the evaluation of susceptibility to bruising. One of them is the total damaged area of the mesocarp. In contrast, we focused on the visible outer layer of the fruit to quantify browning. Since most factories currently use automatic machines for sorting defective olives by color, the parameter of visual color of the skin, which was measured in this study, is the relevant parameter based on which defective drupes are discarded, causing financial losses.

There was significant variation in the different cultivars susceptibility to browning. Some were completely green after impact whereas others were extremely brown several hours after impact. In apples, it has been suggested that variation in bruise size for a given impact energy occurs across cultivars and depends on a number of factors such as maturity and temperature (Samim and Banks, 1993). We tested the cultivars at their “green maturity” stage (table olive harvest time). However, observation of the developmental stage for all cultivars was performed visually and may not always have been accurate. In addition, since each cultivar was tested on a different day, according to its stage of development, the temperature at harvest was not identical. However, the temperature at this season (during September) is fairly stable in Israel. In addition, extreme cultivars were retested, and validation of their susceptibility/resistance to browning was performed. Comparison of the phylogenetic relationships among cultivars analyzed in a previous study (Biton et al., 2015) with the browning pattern and elasticity coefficient of the various cultivars revealed that genetically close cultivars tend to show a similar browning level and elasticity coefficient (**Figure 3**). Therefore, although our analysis may contain some biased results, we are convinced that, in general, and especially for the extreme cultivars, the level of browning presented here for each cultivar is mainly due to genetic differences among cultivars.

### Timing of the Browning Phenomenon

Brown spots appeared on the fruits immediately after the induced pressure and spread over time, until they reached their final size 3 h after pressure was applied. The differences in most cultivars between 3 and 24 h after the induced pressure were not significant. The regression coefficient between the browning proportion at 3 and 24 h after injury was found to be 1.033. This is very close to a regression coefficient of 1, corresponding to equality between the two tested variables. Apart from the three cultivars, “Dolce de Marocain,” “Memecik,” and “Gemlik,” in which the browning process continued after 3 h, in the remaining 103 cultivars, the browning process reached its full extent during the first 3 h after injury. Other studies reported that discoloration continued spreading and reached its final size 24 after the induced pressure (Jiménez-Jiménez et al., 2013a; Jiménez et al., 2016; Jiménez et al., 2017). However, these studies used methods of simulating injury such as dropping the fruits from a height of 1 m to induce damage, whereas we used direct pressure on the fruits. The different methods used could explain the differences in spreading time of the bruising.

The “Manzanillo” cultivar is the most popular table olive in Israel, as well as in other countries (Diego and Rallo, 2000). In this study, the “Manzanillo” showed 5.5% browning 3 h after application of pressure. Other cultivars used as table olives showed resistance to browning, such as “Hojiblanca,” “Kadesh,” “Kadeshon,” “Nabali,” “Niedda de gonnos,” “Picual,” “Sevillano,” “Tonda calliari,” and “Tonda oliana.” Other studies have also shown that there are cultivars more resistant to bruising than the “Manzanillo,” such as “Hojiblanca” (Jiménez et al., 2016) and “Manzanillo Cacerena” (Jiménez et al., 2017). Switching to mechanical harvest of table olives with minimum injury to the harvested fruits can be achieved by replacing sensitive trees with a browning-resistant cultivar.

### A Thick Cuticle Promotes Resistance to Browning

Injuries incurred during mechanical harvesting can be either from blows received from canes used to assist the mechanical shaker in helping the fruit drop from the tree or from fruitshitting the ground after falling from the tree. In both cases, the impact is flat and not sharp. Therefore, we used a force gauge with a flat rounded pinhead to mimic the impact inflicted on the fruit by the mechanical harvester. Not surprisingly, the flat pressure did not damage the exocarp tissue. However, damage was found in the mesocarp layer. Jiménez et al. (2016) also found that no damage was observed in the exocarp as a result of olive fruits falling to the ground. However, mesocarp cells were injured from this treatment. When the penetration force needed to damage the exocarp with a sharp pinhead was measured, significant differences in the resistance of susceptible cultivars were found. Not surprisingly, the susceptible cultivars were characterized by a lower penetration force (**Figure 5**). This validated our presumption that the cuticle of the resistant cultivars may be thicker than that of the susceptible cultivars. It has already been suggested that cuticle thickness is one of the major factors influencing susceptibility to browning (Hammami and Rapoport, 2012). However, this was never tested on several cultivars. A significant difference was found between the cuticle thickness of the cultivars susceptible to browning and that of the resistant cultivars (**Figure 5**). This significant disparity, together with the observation that the exocarp cells remain unharmed after induced pressure even in the susceptible cultivars, while the mesocarp cells were clearly damaged in response to this pressure, suggests that the main factor in determining the difference among cultivars in their susceptibility to browning is the thickness of their cuticle. We used the cuticular flange wedged between epidermal cells to evaluated cuticle thickness. A difference of 31% was found between the average cuticle thickness of the susceptible compare to the resistant cultivars (21.49 and 28.21 µm, respectively). Our results are in agreement with other studies that found thicker cuticles in the more resistant cultivars (Hammami and Rapoport, 2012; Jiménez et al., 2017).

### The Browning Process in Green Olives

Browning of olive fruits after induced injury can be prevented by dipping the fruits in boiling water (**Supplementary Figure 1**).

It was important to ascertain, as suggested by other studies, that the parameter we evaluated—the proportion of brown color on the fruit skin—is the result of an enzymatic process. Several studies have found that dipping the olives in NaOH also inhibits the browning process (Ben-Shalom et al., 1978; Sánchez et al., 2006; Zipori et al., 2014). In plants, PPOs known for their role in post-harvest browning of fruits and vegetables are localized into plastids (Sullivan, 2015). It has also been suggested that this class of enzymes is involved in the browning process of green olives after damage (Ben-Shalom et al., 1978; Sciancalepore and Longone, 1984). PPOs and their potential phenolic substrates have been considered to be physically separated from one another, with most PPOs targeted to the chloroplasts, while phenolic compounds accumulate primarily in the vacuole and cell wall (Vaughn et al., 1988; Steffens et al., 1994). Postharvest browning occurs in damaged plant tissues due to the contact between phenolic compounds and PPOs, leading to transformation of phenolic compounds to colored polymers (Araji et al., 2014; Croguennec, 2016). Based on the results of other studies mentioned above and the conclusions we have reached from our present study, we would like to suggest that the browning process in olives is driven by the PPOs enzymes. We suggest that, as a result of the injury, physical contact between the PPOs and phenolic compounds leads to the brown spots appearing on the surface of the fruit. In the resistant cultivars identified in this study, the mesocarp cells were not harmed by the induced pressure; the PPO and the phenolic compounds remained localized in different cell components, and therefore, no brown spots appeared on the fruits of these cultivars.

## Conclusions

This study describes the variation found in olive cultivars regarding their susceptibility to fruit browning in response to injury occurred during mechanical harvest. The varied browning level as a response to mechanical damage shown by the cultivars could be mainly due to genetic differences among cultivars. Many cultivars can be characterized as browning-resistant due to their thick cuticle. We therefore suggest this simple method of induced pressure described above, in order to screen for new browning-resistant table olive cultivars as the first step in a breeding program aimed at identifying table olive phenotypes suitable for mechanical harvesting.

The two major factors involved in shifting from manual to mechanical harvesting of table olives are harvesting efficiency and prevention of injury to fruit. High harvesting efficiency demands pretreatment which will enable fruit abscission without defoliation. In a recent study, added antioxidants such as ascorbic acid or butyric acid to ethephon spray as a pretreatment to green olive harvest inhibited leaf abscission while enhancing fruit abscission (Goldental-Cohen et al., 2017). In our present study, we have identified 14 cultivars that showed resistance to browning after induced pressure. Among them, nine cultivars are known to produce table olives or to serve as dual purpose. Work is in progress to screen these nine cultivars regarding their response to ethephon and ascorbic acid. Those that respond similarly to “Manzanillo” to ethephon and ascorbic acid then could be used as table olive cultivars suitable for mechanical harvest.

## Data Availability Statement

All datasets generated for this study are included in the manuscript/**Supplementary Files**.

## Author Contributions

SG-C, IB, YM, SB-S, HZ, BA, and GB-A performed the experiments. SG-C and GB-A were involved in data analysis. GB-A wrote the manuscript.

## Funding

This work was supported by the Israeli Ministry of Agriculture and Rural Development (Grant No. 203-0858).

## Conflict of Interest

The authors declare that the research was conducted in the absence of any commercial or financial relationships that could be construed as a potential conflict of interest.

## Supplementary Material

Supplementary Clip Fast-forward play of the three hours following induced pressure, as the browning-sensitive cultivar fruit changes color from green to brown in the area surrounding the point of injury.

Supplementary Figure 1 Illustration of induced pressure by the force gauge, the dipping of fruits in a bath of boiling water and the images taken 3 hours after incubation of the browning-sensitive cultivar ‘Koroneiki’ fruits with and without treatment in boiling water (right and left images, respectively). Boiling the fruits for ten minutes prevented appearance of brown spots on the surface of the fruits.
