# Supplementary figures and images for: Green Olive Browning Differ Between Cultivars
Source: Front Plant Sci. 2019 Oct 8;10:1260. doi: 10.3389/fpls.2019.01260 (PMC6791929; doi:10.3389/fpls.2019.01260)

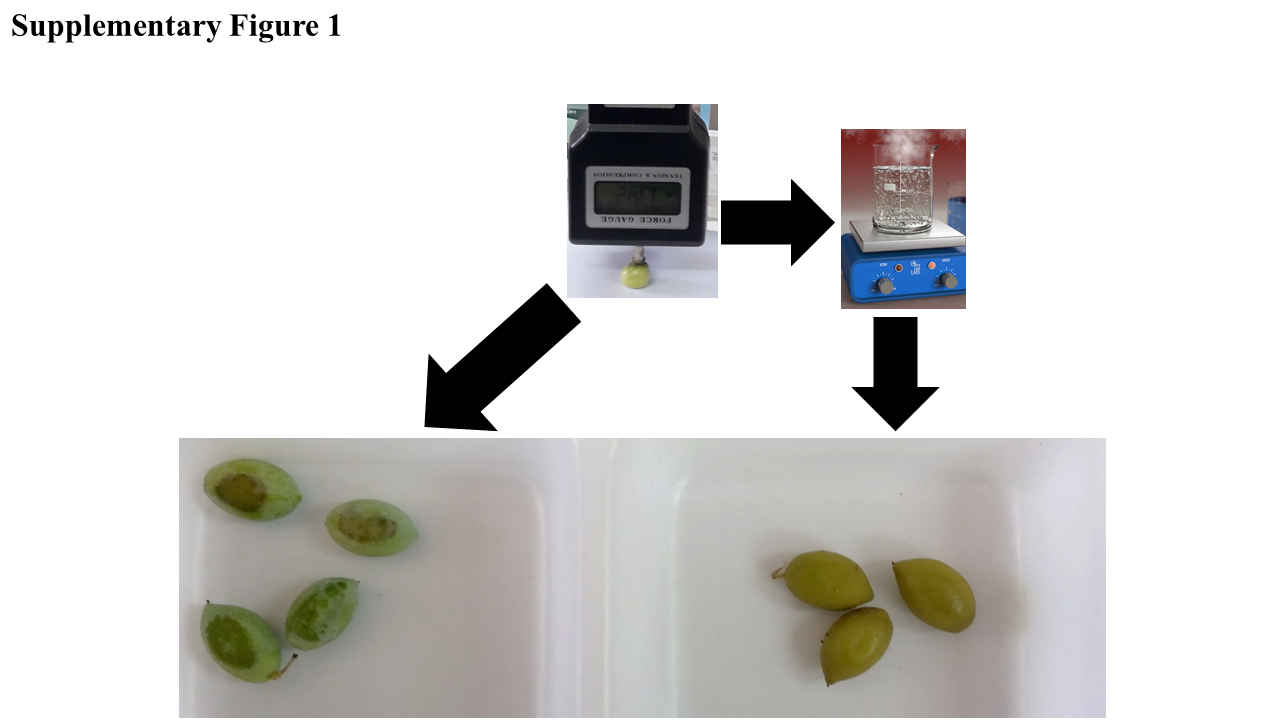

Supplement: Supplementary Figure 1 — Illustration of induced pressure by the force gauge, the dipping of fruits in a bath of boiling water and the images taken 3 hours after incubation of the browning-sensitive cultivar ‘Koroneiki’ fruits with and without treatment in boiling water (right and left images, respectively). Boiling the fruits for ten minutes prevented appearance of brown spots on the surface of the fruits. [file Image_1.tif]
